# Responder analysis for pain relief and numbers needed to treat in a meta-analysis of etoricoxib osteoarthritis trials: bridging a gap between clinical trials and clinical practice

**DOI:** 10.1136/ard.2009.107805

**Published:** 2009-04-12

**Authors:** R A Moore, O A Moore, S Derry, P M Peloso, A R Gammaitoni, H Wang

**Affiliations:** 1 Pain Research and Nuffield Department of Anaesthetics, University of Oxford, Oxford, UK; 2 Department of Rheumatology, Musgrove Park Hospital, Belfast, UK; 3 Merck Research Laboratories, Rahway, New Jersey, USA

## Abstract

**Background:**

Population mean changes from clinical trials are difficult to apply to individuals in clinical practice. Responder analysis may be better, but needs validating for level of response and treatment duration.

**Methods:**

The numbers of patients with pain relief over baseline (⩾15%, ⩾30%, ⩾50%, ⩾70%) at 2, 4, 8 and 12 weeks of treatment were obtained using the WOMAC 100 mm visual analogue pain subscale score for each treatment group in seven randomised placebo-controlled trials of etoricoxib in osteoarthritis lasting ⩾6 weeks. Dropouts were assigned 0% improvement from baseline from then on. The numbers needed to treat (NNTs) were calculated at each level of response and time point.

**Results:**

3554 patients were treated with placebo, etoricoxib 30 mg and 60 mg, celecoxib 200 mg, naproxen 1000 mg or ibuprofen 2400 mg daily. Response rates fell with increasing pain relief: 60–80% experienced minimally important pain relief (⩾15%), 50–60% moderate pain relief (⩾30%), 40–50% substantial pain relief (⩾50%) and 20–30% extensive pain relief (⩾70%). NNTs for etoricoxib, celecoxib and naproxen were stable over 2–12 weeks. Ibuprofen showed lessening of effectiveness with time.

**Conclusion:**

Responder rates and NNTs are reproducible for different levels of response over 12 weeks and have relevance for clinical practice at the individual patient level. An average 10 mm improvement in pain equates to almost one in two patients having substantial benefit.

Clinical trials are performed usually for regulatory purposes, with outcomes typically reported as statistical comparisons between treatment group population means. The results of clinical trials can be difficult to translate into clinical practice. A report that an intervention shows an average 10 mm reduction more than placebo on a 100 mm visual analogue scale has little immediate impact.

Moreover, few of us are average. Most drugs provide a good response in half or fewer of the patients treated,^1^ ^2^ true in postoperative pain,^3^ neuropathic pain,^4^ ^5^ ^6^ migraine^7^ and tumour necrosis factor antagonists in rheumatoid arthritis.^8^ An 80/20 rule seems to apply in osteoarthritis, with 80% of patients experiencing 20% pain relief but only 20% experiencing 80% relief; about half have their pain halved.^9^

Genetic influences help determine the clinical response to analgesic drugs for non-specific anti-inflammatory drugs (NSAIDs),^10^ opioids^11^ and more generally,^12^ as well as the clinical response to methotrexate.^13^ Pain is driven by complex pathways of neural mechanisms which are likely to be different between individuals.^14^ Imaging reveals loss of grey matter in chronic pain above that found with age alone.^15^ ^16^

Average data from skewed distributions can produce misleading results.^17^ Dichotomous responder analyses have been reported previously for acute^18^ and chronic pain.^5^ ^6^ ^19^ The validity of a dichotomous measure should be established before being widely used.^20^

An added factor contributing to differences in treatment response observed in clinical practice compared with a clinical trial is the handling of dropouts. Commonly, a “last observation carried forward” technique is used in clinical trials, where data from patients with good pain control but intolerable adverse events will still be included in efficacy calculations using the population mean. In clinical practice, this same patient would be considered a treatment failure.

We used individual patient data from seven randomised placebo-controlled trials in osteoarthritis to investigate the effects of different levels of pain relief assessed at various time points on estimates of efficacy.

## Methods

Merck Research Laboratories provided pain response data from seven randomised placebo-controlled trials of etoricoxib in osteoarthritis lasting ⩾6 weeks (protocols 007, 018, 019, 071, 073, 076 and 077).^21^ ^22^ ^23^ ^24^ ^25^ ^26^ PDF copies of the company clinical trial reports were also available.

We calculated the number of patients in each treatment group in each trial achieving various Initiative on Methods, Measurement, and Pain Assessment in Clinical Trials (IMMPACT) thresholds of pain relief over baseline of ⩾15% (minimal benefit), ⩾30% (moderate), ⩾50% (substantial)^27^ and ⩾70% which we defined as extensive improvement. These were assessed at 2, 4, 8 and 12 weeks. All trials lasted 12 weeks except protocol 007 which lasted 6 weeks.

In each study patients were asked, “During the last 48 hours, how much pain do you have (1) walking on a flat surface; (2) going up or down stairs; (3) at night while in bed; (4) sitting or lying; (5) standing upright?”. On a 100 mm visual analogue scale, patients placed an “x” ranging from 0 (“no pain”) to 100 (“extreme pain”). The Western Ontario and McMasters Universities (WOMAC) 100 mm visual analogue pain subscale score was calculated as the average of the responses to the five questions.

Criteria used in defining responders included:

- For patients who did not drop out, only actual measured values were used for calculations. Last observation carried forward was not used.
- For patients who withdrew for any reason, measurements made within 7 days of the last dose were used to calculate the response.
- Thereafter, patients were assigned 0% improvement.

We calculated the number and percentage of responders for each level of response for each drug and time point and the number needed to treat (NNT) compared with placebo (with 95% CI).^28^ The relative risk with 95% CI was calculated using the fixed effects model^29^ and considered statistically significant when the 95% CI did not include 1. Statistically significant differences between NNTs were established using the z test,^30^ comparing different drug/dose combinations only in the trials in which they were used together.

Clinical trial reports were used to obtain, for each active treatment, the difference between active treatment and placebo for the WOMAC pain subscale score. This was defined in the company clinical trial reports as the mean time-weighted average change from baseline (flare/randomisation visit) over the 6- or 12-week treatment period ((WOMAC baseline − WOMAC treatment average) − (WOMAC baseline − WOMAC placebo average)). Average results for each treatment arm were pooled using RevMan 5.0.

## Results

Information was available on 3554 patients (two-thirds women) with an average age of 62 years (see online supplement). Six trials involved patients with osteoarthritis of the knee or hip and one of the knee only. Osteoarthritis was established clinically and radiographically. Initial pain had to be a minimum of 40/100 mm at inclusion, plus ⩾15 mm increase and worsening in investigator global assessment since baseline. The actual numbers of responders for each level of response for each drug in each trial and at each time point are shown in the online supplement.

**Figure 1:**
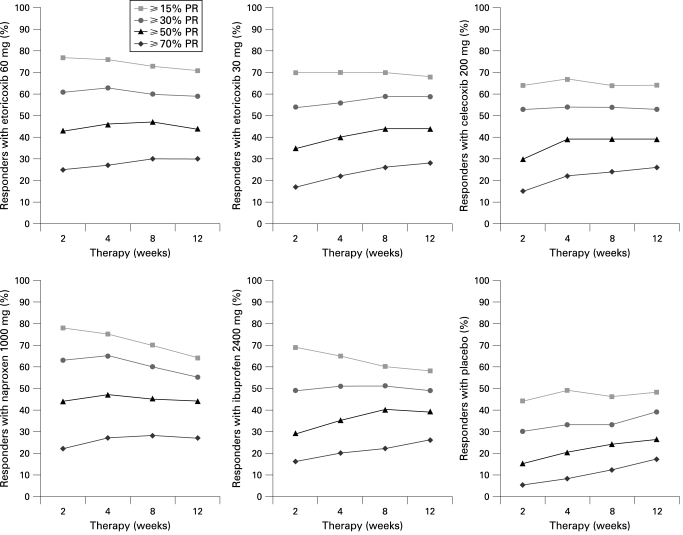
Percentage of responders over baseline at various levels of reductions in pain intensity (PR) for placebo and five active drugs over 12 weeks of treatment.

**Figure 2:**
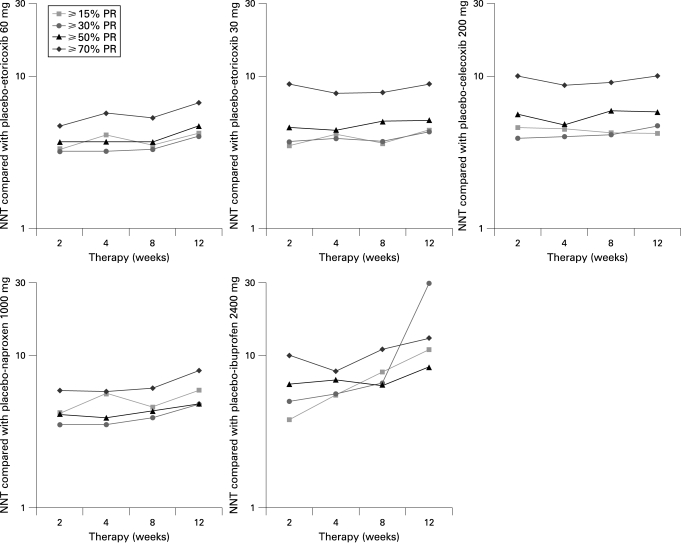
Numbers needed to treat (NNT) compared with placebo for five active drugs over 12 weeks of treatment using various levels of reductions in pain intensity (PR) over baseline.

Table 1 gives the percentage of responders and NNTs for each level of response for each drug in each trial and at each time point. The percentage of responders and NNTs are also shown in figs 1 and 2, respectively.

**Table 1:** Percentage of responders with treatment/placebo and numbers needed to treat (NNTs) after 2, 4, 8 and 12 weeks

Outcome	Percentage of responders with treatment/placebo after different numbers of weeks				NNT (95% CI)			
	2	4	8	12	2	4	8	12
Etoricoxib 30 mg; 5 trials, 1486 patients								
⩾15%	70/41	70/45	70/43	68/45	3.5 (2.9 to 4.2)	4.1 (3.4 to 5.2)	3.6 (3.0 to 4.4)	4.4 (3.5 to 5.7)
⩾30%	54/27	56/30	59/31	59/36	3.7 (3.1 to 4.5)	3.9 (3.3 to 4.9)	3.7 (3.1 to 4.5)	4.3 (3.5 to 5.6)
⩾50%	35/13	40/18	44/23	44/24	4.6 (3.8 to 5.6)	4.4 (3.7 to 5.5)	5.0 (4.0 to 6.5)	5.1 (4.1 to 6.9)
⩾70%	17/5	22/9	26/13	28/17	8.9 (7.0 to 12)	7.7 (6.0 to 11)	7.8 (6.0 to 11)	8.9 (6.3 to 15)
Etoricoxib 60 mg; 3 trials, 711 patients								
⩾15%	77/46	76/51	73/44	71/47	3.3 (2.6 to 4.5)	4.1 (3.1 to 6.2)	3.5 (2.7 to 4.9)	4.2 (2.9 to 7.4)
⩾30%	61/31	63/32	60/30	59/35	3.2 (2.6 to 4.6)	3.2 (2.6 to 4.4)	3.3 (2.6 to 4.5)	4.0 (2.9 to 6.7)
⩾50%	43/16	46/19	47/20	44/23	3.7 (2.9 to 5.0)	3.7 (2.9 to 5.1)	3.7 (2.9 to 5.1)	4.7 (3.3 to 8.1)
⩾70%	25/4	27/9	30/11	30/15	4.7 (3.9 to 6.0)	5.7 (4.3 to 8.4)	5.3 (4.0 to 7.7)	6.7 (4.4 to 14)
Celecoxib 200 mg; 2 trials, 714 patients								
⩾15%	64/42	67/45	64/40	64/40	4.6 (3.4 to 7.0)	4.5 (3.3 to 6.7)	4.2 (3.2 to 6.1)	4.2 (3.2 to 6.1)
⩾30%	53/27	54/29	54/30	53/31	3.9 (3.0 to 5.3)	4.0 (3.1 to 5.6)	4.1 (3.2 to 5.9)	4.7 (3.5 to 7.1)
⩾50%	30/12	39/18	39/22	39/22	5.6 (4.2 to 8.3)	4.8 (3.6 to 6.9)	5.9 (4.2 to 9.9)	5.8 (4.2 to 9.5)
⩾70%	15/6	22/10	24/13	26/16	10 (7.1 to 18)	8.7 (5.9 to 16)	9.1 (6.0 to 19)	10 (6.3 to 27)
Naproxen 1000 mg; 2 trials, 531 patients								
⩾15%	78/54	75/57	70/48	64/47	4.2 (3.0 to 7.5)	5.6 (3.6 to 13)	4.6 (3.1 to 8.8)	5.9 (3.6 to 15)
⩾30%	63/35	65/36	60/35	55/35	3.5 (2.6 to 5.5)	3.5 (2.6 to 5.4)	3.9 (2.8 to 6.4)	4.8 (3.2 to 9.2)
⩾50%	44/20	47/22	45/22	44/23	4.1 (3.0 to 6.3)	3.9 (2.9 to 6.0)	4.3 (3.1 to 7.0)	4.8 (3.3 to 8.5)
⩾70%	22/5	27/10	28/12	27/15	5.9 (4.4 to 8.8)	5.8 (4.1 to 9.7)	6.1 (4.2 to 11)	8.0 (4.9 to 21)
Ibuprofen 2400 mg; 2 trials, 618 patients								
⩾15%	69/43	65/47	60/48	58/49	3.8 (2.9 to 5.5)	5.5 (3.8 to 10)	7.8 (4.8 to 22)	11 (5.8 to 121)
⩾30%	49/29	51/33	51/36	49/41	5.0 (3.6 to 8.1)	5.6 (3.9 to 10)	6.6 (4.3 to 14)	NS
⩾50%	29/14	35/20	40/24	39/27	6.5 (4.6 to 11)	6.9 (4.6 to 14)	6.4 (4.3 to 12)	8.4 (5.1 to 24)
⩾70%	16/6	20/8	22/13	26/18	10 (7.0 to 21)	7.9 (5.6 to 14)	11 (6.6 to 32)	13 (6.8 to 75)

Response with placebo is for placebo groups from trials in the particular comparison being made.

The percentage of patients achieving levels of pain relief with placebo at each threshold rose between weeks 2 and 12 (table 1). At the end of 12 weeks the proportion of patients was about 45% for a response of ⩾15%, 35% for ⩾30%, 25% for ⩾50% and 15% for ⩾70%. More patients achieved each level of response with active drug than with placebo (table 1, fig 1). Etoricoxib 30 mg and 60 mg daily and celecoxib 200 mg daily had similar response patterns, with constant percentages at lower response levels but a tendency over time for the proportion achieving ⩾70% pain relief by 12 weeks to increase. Naproxen 1000 mg daily and ibuprofen 2400 mg daily had different response patterns, with stable or increasing percentages at higher levels (⩾50%, ⩾70%) but progressive decreases in the percentage achieving lower levels of response of ⩾15% and ⩾30%.

NNTs calculated for ⩾15%, ⩾30% and ⩾50% pain relief were very similar with etoricoxib 60 mg and 30 mg, celecoxib 200 mg and naproxen 1000 mg (table 1, fig 2). NNT values were between 3 and 5 over the 12 weeks of measurement. For ⩾70% pain relief the NNT was distinctly higher with values between 6 and 10. The pattern for ibuprofen 2400 mg was different, with NNTs generally much higher (worse) at longer study duration and less consistency between the various levels of response.

There were three direct comparisons, each in two trials, of daily doses of etoricoxib 60 mg and naproxen 1000 mg, etoricoxib 30 mg and celecoxib 200 mg, and etoricoxib 30 mg and ibuprofen 2400 mg. No statistically significant differences were found between them at any level of response or any duration of treatment.

**Figure 3:**
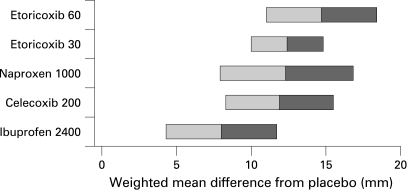
Weighted mean difference between treatment and flare/randomisation visit for WOMAC pain subscale: active treatment minus placebo. Shading shows upper and lower 95% confidence intervals.

The additional reduction in the WOMAC pain subscale score for each active treatment over placebo between the flare/randomisation visit and end of treatment is shown in fig 3. The smallest mean difference above placebo was 8 mm for ibuprofen 2400 mg daily and the largest was 15 mm for etoricoxib 60 mg daily. This shows that about 60% of patients have moderate benefit (fig 1) while average reductions in pain over placebo appear modest.

## Discussion

Population mean changes have no easy resonance outside a clinical trial. An average of 10 mm out of 100 mm (10% improvement; fig 3) conveys no expectation of great benefit, with little to balance against known risks. A different approach is needed.

A pilot study of responder analysis in a single trial indicated that it might be a useful way of reporting pain results in osteoarthritis.^9^ It suggested that at least 50% pain relief after 6 weeks of treatment could be a useful discriminator between interventions of greater and lesser efficacy. This needs validation with regard to different levels of pain relief, and especially duration, given that arthritis treatments are used in the medium to long term.

IMMPACT provided recommendations for interpreting the clinical importance of treatment outcomes in clinical trials of chronic pain.^27^ It suggested that a 10–20% decrease in pain intensity was minimally important, ⩾30% moderately important and ⩾50% substantial. This meta-analysis used these three discriminator points, together with the even higher discriminator of ⩾70% pain relief, to perform a responder analysis, calculate NNTs and examine the effects of trial duration.

Response rates declined as the discriminator level increased for all five active drugs and placebo (fig 1, table 1). Using the IMMPACT descriptions for commonly used NSAIDs, 60–80% of patients with osteoarthritis can expect minimally important pain relief, 50–60% moderate pain relief, 40–50% substantial pain relief and 20–30% extensive pain relief.

With placebo and active drugs the proportion achieving higher levels of pain relief increased over time, perhaps due to a natural waning of pain inherent in the “flare” design. The tendency to less response at lower levels of pain relief over time with naproxen and ibuprofen may reflect higher withdrawal rates for traditional NSAIDs over cyclooxygenase-2 selective inhibitors.^31^ ^32^ Patients may be balancing benefit and harm, with lower levels of relief perhaps not worthwhile in the face of adverse events. In the responder approach, dropouts contribute to the denominator only, whereas with “last observation carried forward” they appear to continue to benefit after withdrawal.

NNTs were comparable for pain relief of ⩾15%, ⩾30% and ⩾50%, with higher (worse) NNTs for ⩾70% for both doses of etoricoxib, celecoxib and naproxen. For these three drugs, NNTs were reasonably stable over 2–12 weeks. Ibuprofen 2400 mg daily was different, with NNT values generally increasing (worsening) with longer duration. The longitudinal responder analysis provides more insight than population average differences (fig 3). This apparently different behaviour with ibuprofen 2400 mg daily did not translate into a statistically significant difference in NNTs between ibuprofen and etoricoxib 30 mg, nor was a significant difference found between etoricoxib 30 mg and celecoxib 200 mg, or etoricoxib 60 mg and naproxen 100 mg. Establishing a dose-response in analgesic trials is known to be difficult even where direct comparisons are available in relatively simple models such as postoperative pain.^33^ Demonstrating an absolute difference in response of 10% requires a substantial number of trials and patients. Confirming statistically that ibuprofen 2400 mg is inferior to other NSAIDs at commonly used doses in osteoarthritis will require more data.

A range of ±0.5 NNT was proposed to determine whether an NNT has “clinical relevance”—whether the NNT is within acceptable bounds of clinical “accuracy”.^34^ A subsequent proposal was that ±0.5 NNT could be used to determine that NNTs were different.^27^ If a numerical difference between NNTs of 1 (eg, 3.5 vs 5.0) was taken to be important, then the application to NNTs in table 1 would begin to differentiate between drugs, with ibuprofen particularly being judged less effective.

The results of the responder analysis achieve the same global conclusions as the original trials—namely, that etoricoxib and its comparators have useful analgesic properties in osteoarthritis. Arguably the most important outcome from these analyses is that, for osteoarthritis, patients and professionals can be provided with trial data that translate into clinical practice by using realistic estimates of the chance of achieving a particular level of benefit. For pain, a mean difference of 10 mm over placebo translates into about 40% having substantial benefit and 30% not having even minimal benefit. Most people with osteoarthritis treated with an NSAID at an appropriate dose can expect to get at least a minimal benefit (though 1 in 5 will not), almost 1 in 2 can expect a substantial benefit and about 1 in 5 an extensive benefit. The prospect of a 1 in 2 chance of substantial benefit has considerably more impact than an average 10% improvement in pain. Moreover, the information is conveyed in terms of both the likelihood of benefit (1 in 2) and the extent of the benefit (substantial).

Patients and professionals are interested in the known associated risks of both common and rare harm. These, too, can be conveyed in terms of likelihood of harm and its consequences.^35^ Balancing benefit and harm is easier when common language describes both. Providing information about the chance of response at various thresholds might produce a more realistic appreciation of benefits and risks of treatment. There is, of course, the caveat that these results come from flare designs in clinical trials where patient selection criteria may make the population different (less comorbidity, perhaps) than a clinical practice population.

Responders are defined not just by the level of response but by the outcome used to define response. We chose the WOMAC pain subscale, combining pain associated with walking, climbing stairs, sitting, lying down and at night while in bed. Whether pain is the most appropriate outcome for responder analysis or whether function, sleep, quality of life or compound outcomes like the OMERACT-OARSI set of responder criteria^36^ are preferable remains to be examined. Finally, this type of analysis may differentiate between treatments in a manner that is not possible with population mean changes in pain intensity, and discriminatory power may reside in different outcomes.

Responder analysis looks promising and much more helpful than an average change of a few millimetres based on populations of responders combined with non-responders.

## Conclusion

Population mean change in pain intensity reported in clinical trials may be difficult to translate into clinical decision-making and patient expectations of benefit. Responder analyses and NNTs calculated from them are reproducible for different levels of response and over at least 12 weeks of treatment with effective drugs. This offers the possibility of providing patients and professionals with information on the chance of achieving particular degrees of pain relief, improving clinical decision-making and patient communication.
